# BATF and BATF3 deficiency alters CD8+ effector/exhausted T cells balance in skin transplantation

**DOI:** 10.1186/s10020-024-00792-0

**Published:** 2024-01-31

**Authors:** Chenghao Li, Zongtao Liu, Zihao Wang, Wai Yen Yim, Yajun Huang, Yuqi Chen

**Affiliations:** 1 Department of Cardiovascular Surgery, Union Hospital, Tongji Medical College, Huazhong University of Science and Technology, Wuhan, China; 2 Department of Plastic Surgery, Xiangyang Central Hospital, Affiliated Hospital of Hubei University of Arts and Science, 136 Jingzhou Street, Xiangyang, Hubei China

**Keywords:** CD8^+^ T-cells, transcription factors, BATF, Graft rejection

## Abstract

**Background:**

It is well-established that CD8^+^ T-cells play a critical role in graft rejection. The basic leucine zipper ATF-like transcription factor (BATF) and BATF3 are transcriptional factors expressed in T lymphocytes. Herein, we investigated the functions of BATF and BATF3 in the differentiation and exhaustion of CD8^+^ T cells following alloantigen activation.

**Methods:**

Wild-type CD8^+^ T cells, BATF-deficient (*Batf*^−/−^) CD8^+^ T cells, and CD8^+^ T cells deficient in both BATF and BATF3 (*Batf*^−/−^*Batf*3^−/−^) were transferred to B6.*Rag1*^−/−^ mice, which received skin allografts from BALB/c mice. Flow cytometry was conducted to investigate the number of CD8^+^ T cells and the percentage of effector subsets.

**Results:**

BATF expression positively correlated with effector CD8+ T cell differentiation. BATF and BATF3 deficiency promoted skin allograft long-term survival and attenuated the CD8+ T cell allo-response and cytokine secretion. Finally, BATF and BATF3 deficiency prompted the generation of exhausted CD8+ T cells.

**Conclusions:**

Overall, our findings provide preliminary evidence that both BATF and BATF3 deficiency influences the differentiation of effector CD8+ T cells and mediates the exhaustion of CD8^+^ T cells, prolonging transplant survival. Targeting BATF and BATF3 to inhibit CD8^+^ T cell function has huge prospects for application as a therapeutic approach to prevent transplant rejection.

**Supplementary Information:**

The online version contains supplementary material available at 10.1186/s10020-024-00792-0.

## Introduction

It has been established that graft rejection, the main factor responsible for graft loss, involves multiple mechanisms. Current evidence suggests that CD8^+^ T cells, an important immune system member, promote transplant rejection through cytotoxic effects (Russell and Ley 2002). During transplant rejection, CD8^+^ T cells can be activated through direct or indirect pathways to produce pro-inflammatory cytokines, and cytotoxic molecules enhance graft rejection (Benichou et al. 1999; Siu et al. 2018).

In addition to the direct alloantigen recognition, recipient naïve CD8^+^ T cells can be activated by the alloantigen presented by CD4^+^ T cell-permitted Dendritic cells after transplantation (Harper et al. 2015). Interestingly, activated naïve CD8^+^ T cells differentiate into effector T cells, secrete cytokine and exert cytotoxic effects. But when the antigen cannot be cleared, differentiation of CD8+ T cells follows another program. Under persistent antigenic stimulation, inhibitory receptors are highly expressed, and CD8+ T cells retain suboptimal but crucial functions to limit progression but cannot eradicate the antigen (Blank et al. 2019). This kind of state was regarded as exhaustion T cells.

The basic leucine zipper ATF-like transcription factor (BATF) family, composed of BATF (also known as SFA2), BATF2 (also known as SARI), and BATF3 (also known as JDP1 and p21SNFT), belongs to the group of activator protein 1 (AP-1) family transcription factors (Murphy et al. 2013). In the immune system, BATF and BATF3 are expressed in T lymphocytes and work synergistically (Hildner et al. 2008). It has been established that BATF and BATF3 mediate the function and differentiation of T cells through their AP-1 control feature, which prevents the cooperation between Activator protein 1 and IFN Regulatory Factor to control gene transcription (Li et al. 2012).

On the one hand, in a mouse infection model, BATF has been shown to influence CD8^+^ T cell responses, and its deficiency leads to an altered induction of antiviral CD8^+^ T effector cells (Chen et al. 2021; Grusdat et al. 2014). Meanwhile, it has been confirmed that BATF is a critical regulator in mediating exhausted CD8^+^ T cells transition in chronic infection (Boi and Lan 2021). On the other hand, BATF3 can regulate the function of CD8^+^ T cells through direct or indirect pathways. BATF3-deficient CD8^+^ T cells have been reported to exhibit defective T cell memory formation (Ataide et al. 2020), and early activation of CD8^+^ T cells is impacted by the cross-presentation of BATF3-dependent cDC (Hildner et al. 2008). Intriguingly, a certain degree of cross-regulation between BATF and BATF3 has been reported. In this respect, high levels of BATF3 were observed when BATF expression was inhibited and complemented the lack of BATF in terms of function (Ise et al. 2011; Wang et al. 2022; Yang et al. 2016). Nevertheless, the study about the deficiency of both BATF and BATF3 is rare. In the field of transplantation, previous work showed BATF and BATF-deficient mice lost the ability to reject the heart allograft with impaired function of effector CD4^+^ T cells; however, the role of BATF and BATF3 during CD8^+^ T cells has not yet been fully elucidated. Herein, the mouse skin transplantation model was used to explore if BATF family member BATF and BATF3 will regulate effector/exhaustion CD8^+^ T cell formation.

## Materials and methods

### Mice

B6.*Rag1*^−/−^ mice, *Batf*^−/−^ (B6.129S-*Batf*^tm1.1 kmm^/J), and *Batf*3^−/−^ (B6.129 S(C)-*Batf3*^tm1Kmm^/J) mice were purchased from the Jackson Laboratory (Bar Harbor). *Batf*^−/−^*Batf*3^−/−^ double-deficient mice were generated by crossing *Batf*^−/−^ mice with *Batf*3^−/−^ mice. C57BL/6 (B6), BALB/c mice were purchased from Gempharmatech company (Nanjing, China). All animals were bred and raised in a specific pathogen-free conditions at Tongji Medical College, Huazhong University of Science and Technology, Wuhan, China.

### In vitro T-cell stimulation

Splenocytes were isolated from wild-type (WT) B6 mice, CD8^+^ T cells were isolated by using the Dynabeads Untouched Mouse CD8 Cells Kit (Thermo Fisher Scientific), seeded in 96-well round-bottom culture plates (3 × 10^5^ cells/well), and cultured with 2 µg/mL soluble anti-CD3e mAb (clone 145-2C11, BioLegend) and 1 µg/mL soluble CD28 (clone 37.51, BioLegend) for 24 h. Stimulated CD8^+^ T cells were analyzed using an LSR II or Fortessa flow cytometer (BD Biosciences).

### CD8+ T cell adoptive transfer model building

CD8^+^ T cells were isolated from the spleens of WT B6 mice and *Batf*^−/−^ *Batf3*^−/−^ mice using the Dynabeads Untouched Mouse CD8 Cells Kit (Thermo Fisher Scientific). The purified CD8^+^ T cells reached over 95% (Additional file 1: Figure S1). Half million CD8^+^ T cells were injected into *Rag1*^−/−^ mice intravenously (i.v.) on day − 1 after injection. BALB/c skin were transplanted into *Rag1*^−/−^ mice on day 0.

### Skin transplantation

Full-thickness tail skin (~ 1 cm^2^) transplantation from BALB/c mice into B6. *Rag1*^−/−^ mice was performed as previously described (Pakyari et al. 2016). Graft rejection is defined as over 90% necrosis (wrinkled blackened skin) of the donor skin tissues. The graft rejection level was verified in the skin allografts harvested on day 14 using hematoxylin and eosin staining. Skin rejection was scored based on Parenchymal rejection (PR) score. Details are as follows: 0 (no rejection), 25 (focal mononuclear cell infiltrates without necrosis), 50 (focal mononuclear cell infiltrates with necrosis), 75 (multifocal infiltrates with necrosis), and 100 (widespread infiltrates with vasculitis).

### Tissue histology

Allografts from recipients were harvested and fixed in formalin. The skin tissues were paraffin-embedded and stained with hematoxylin and eosin at the Cardiac surgery laboratory of Union Hospital (Tongji Medical College, Huazhong University of Science and Technology). Tissue sections were evaluated under a light microscope (Nikon Eclipse 80i; Nikon).

### RNA-seq analysis

CD8^+^ cells isolated from WT mice were stimulated with anti-CD3e and CD28 for 0 h or 24 h, then stored in TRIzol (Thermo Fisher Scientific). The SMART seq v4 kit lib (Takara Inc.) was used to send the samples to Novogene Inc. for low-input total RNA library construction. Genes with an RNA integrity number (≥ 8.0) were further sequenced on the Illumina HiSeq platform. We then obtained an average insert size of 150 bp paired-end libraries. Then, the in-house pipeline from Novogene was used to evaluate the GC content and error rate. Raw reads were aligned to the mouse reference (mm^10^) using the STAR aligner (version 2.6). Finally, differential gene expression analysis was performed using the DESeq2 R package. A de model based on a negative binomial distribution (log2 [fold change]) was used to determine differential gene expression. Genes were annotated using R package cluster Profiler by KEGG pathway enrichment analysis of DEGs, and the pathways enriched for these genes were analyzed.

### Flow cytometry analysis

Cells were first prepared from the spleen or drained lymph nodes (dLNs) and then stained with fluorochrome-conjugated antibodies specific for mouse CD45 (30-F11), CD8 (53–6.7), CD62L (MEL- 14), CD44 (IM7), TCRβ (H57–597), killer cell lectin-like receptor G1 (KLRG1) (2F1/KLRG1), IL- 2 (JES6-5H4), IFN-γ (XMG1.2), TNF-α (MP6-XT22), CD279 (PD-1) (29F.1A12), granzyme B (GzB) (QA16A02), Ki67 (16A8), CD39 (Duha59), CD223 (LAG-3) (C9B7W), IRF4 (IRF4.3E4), T-bet (4B10), BATF (9B5A13) and Helios (22F6) before or after CD8^+^ T cells isolation,. All antibodies were purchased from BioLegend. Antibodies against TOX (TXRX10), Id2 (ILCID2), CD127 (ebioSB/199) and CXCR3 (CXCR3- 173) were purchased from Thermo Fisher Scientific. Antibody against TCF-1 (C63D9) were purchased from Cell Signaling Technology. For surface markers, cells were first collected from the spleen or dLNs, followed by passing through a 70 µm strainer. Next lysed erythrocyte, resuspended into cell suspensions and stained with fluorochrome-conjugated Abs according to the manufacturers' instructions. For intracellular staining, cells were fixed and permeabilized with Foxp3/Transcription Factor Staining Buffer Set (00-5523-00) from Thermo Fisher Scientific according to the manufacturer’s instructions. For intracellular secretory cytokine staining, cells were briefly restimulated for 4 h with Cell Activation Cocktail (with Brefeldin A)(BioLegend), and then fixed and permeabilized with Foxp3/Transcription Factor Staining Buffer Set and stained with fluorochrome-conjugated Abs according to the manufacturers’ instructions.

### Statistical analysis

Data are represented as mean ± standard deviation and were analyzed with Prism version 8.0 (GraphPad Software). The p-values of skin and heart graft survival were plotted using the Mann–Whitney test. Other measurements were analyzed using an unpaired Student’s *t*-test. Differences were considered as follows: not significant, p > 0.05; *p < 0.05; **p < 0.01, ***p < 0.001.

## Results

### BATF expression is positively correlated with effector CD8+ T cell differentiation

**Fig. 1:**
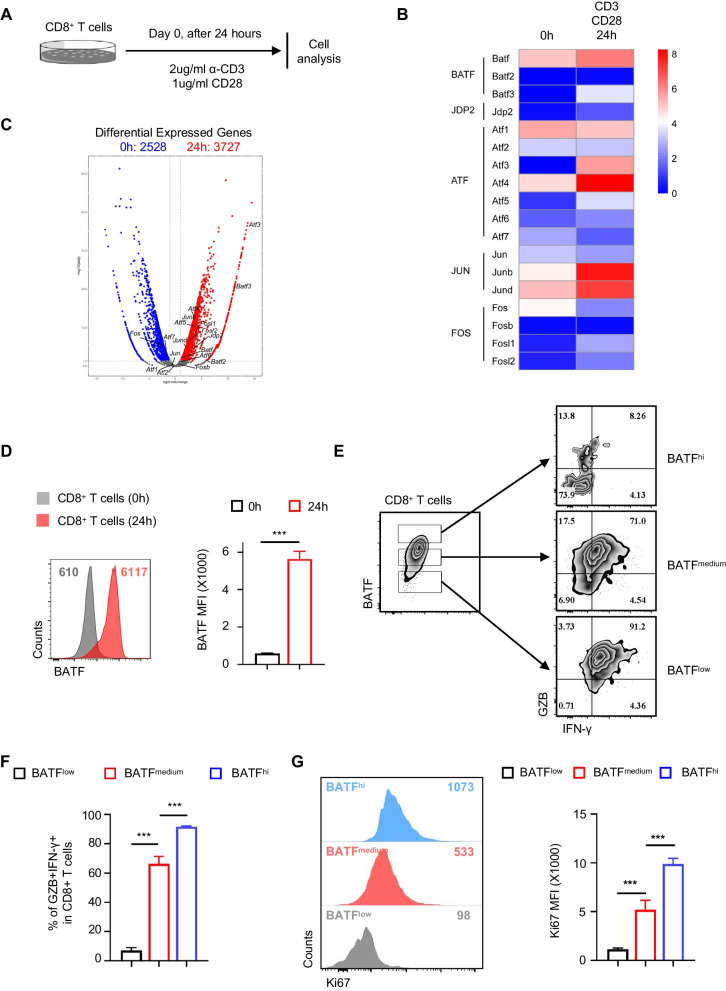
BATF expression is positively correlated with effector CD8^+^ T cell differentiation. **A** Splenocytes from WT B6 mice were stimulated with 2 µg/ml soluble anti-CD3e mAb and 1µg/ml soluble CD28 for 24 h, followed by RNA- seq analysis and flow cytometric analysis. One WT B6 mice were used for isolated CD8+ T cells and stimulation of CD8+ T cells. **B** The volcano plot shows up- and down- regulated genes in 0h stimulated CD8+ T cells and 24h stimulated CD8+ T cells (red dots). The STAR program was used to align the reads to the mouse reference file (GENCODE mouse reference GRCm38). R package (edgeR) was used to analyze differential expression with raw read count of each gene adjusted by trimmed mean of M values (TMM). The significant differentially expressed genes are both padjust < 0.05 and |log2(FoldChange)|> 1. **C** Heatmap based on FPKM of selected differentially expressed genes. Color is proportional to the relative abundance of gene expression cross sample, that is blue stands for low expression and red stands for high expression. **D** The graph shows Batf MFI of CD8+ T cells. **E**, **F** Representative contour plots and bar graphs show the expression of IFN-γ and granzyme B in CD8+ T-cell subpopulations that express high, medium or low levels of batf. **G** Counts and MFI in three batf repressed CD8+ T-cell subpopulations. Data are mean ± SD (n = 5). **p < 0.01, ****p < 0.0001 (unpaired Student’s t-test)

To determine the role of the BATF family in CD8^+^ T cell differentiation and function, we detected BATF family expression in CD8^+^ cells before and after activation by flow cytometry and cell analysis. Splenocytes from wild-type B6 mice were detected using flow cytometry after stimulation with 2 µg/mL soluble anti-CD3e mAb and 1 μg/mL CD28 for 24 h (Fig. 1A). As shown in Fig. 1B, C, compared to control CD8^+^ T cells, activated CD8^+^ T cells expressed upregulated levels of AP-1 family genes, such as *Batf*, *Batf3*, *Atf3*, *Atf4*, and *Jdp2*. Meanwhile, some members of the Jun and Fos families, such as *Junb*, *Jund*, *Fosl1*, and *Fosl2*, were upregulated in activated CD8^+^ T cells, while Jun and Fos expression levels were downregulated.

Flow cytometry analysis showed that the expression of BATF in activated CD8^+^ T cells was significantly higher than in CD8^+^ T cells without α-CD3 and CD28 stimulation (Fig. 1D, p < 0.001). To understand the relationship between BATF and the function of CD8^+^ T cells, we examined the association between BATF expression and the secretion of the functional cytokines GzB and IFN-γ from CD8 cells. Flow cytometry showed that most CD8^+^ T cells with moderate or high BATF expression exhibited high GzB and IFN expression, while CD8^+^ T cells with low BATF expression hardly produced GzB and IFN-γ (Fig. 1E, F, p < 0.001). Interestingly, we found that BATF expression was positively correlated with Ki67 expression in CD8^+^ T cells (Fig. 1G, p < 0.001). These phenomena suggest that BATF is closely associated with the proliferation and effector functions of CD8^+^ T cells.

### BATF and BATF3 deficiency promotes long-term skin allograft survival

**Fig. 2:**
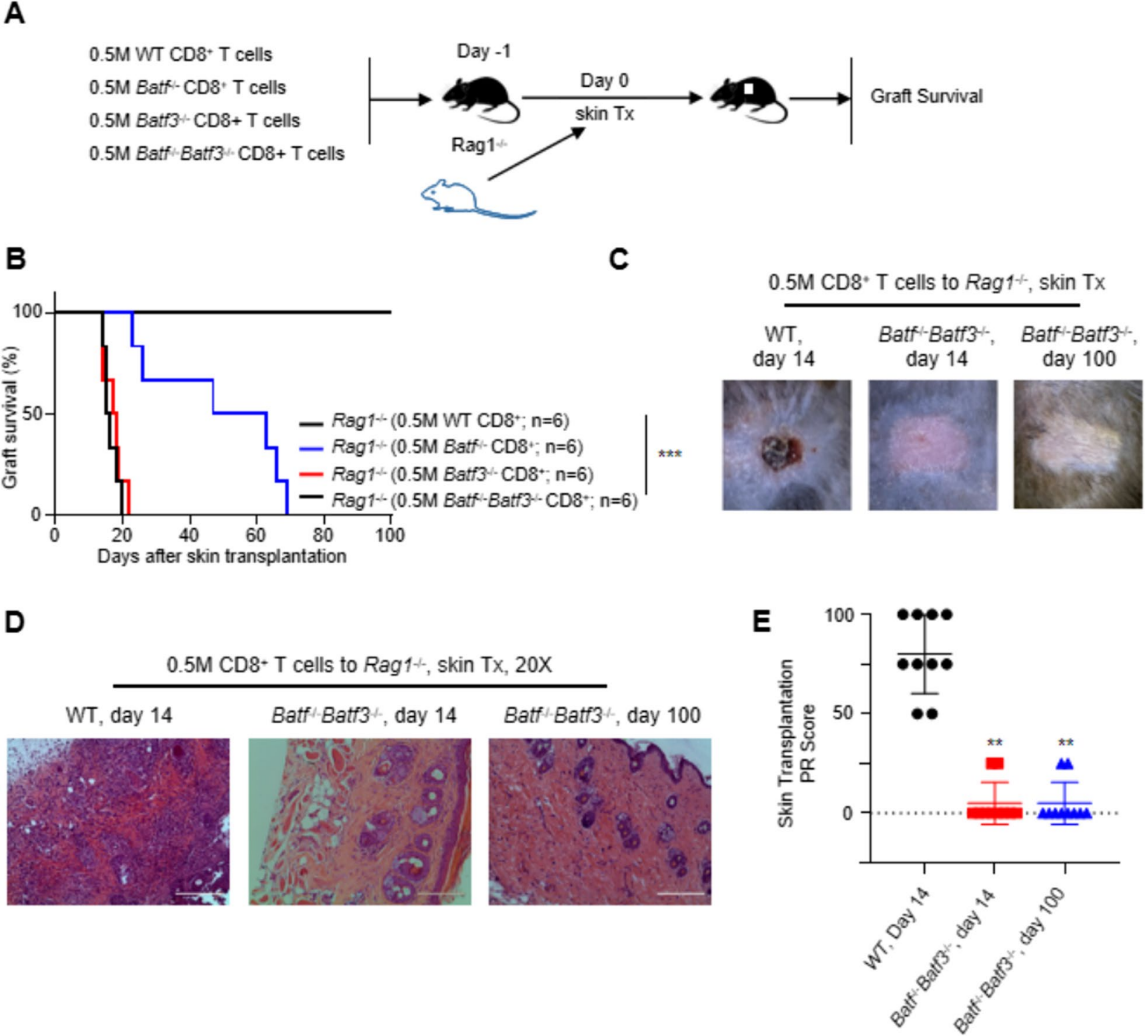
BATF and BATF3 deficiency promotes long-term skin allograft survival. **A** Experimental design involving lymphopenic B6.Rag1−/− recipients adoptively co-transferred with equal numbers of WT CD8+ T cells, Batf−/− CD8+ T cells and Batf−/− Batf3−/− CD8+ T cells and grafted with BALB/c skin allografts. Tx, transplantation. **B** The percentage of graft survival post-allogeneic tail skin transplantation (n = 6). ***p < 0.001; Mann–Whitney test. **C**, **D** Representative images and H&E- stained images of rejected and accepted tail skin allografts posttransplant corresponding to (**B**). **E** PR scores of allograft tissue sections were evaluated on days 14 and days 100 after transplantation. **p < 0.01;***, p < 0.001

To investigate the role of BATF and BATF3 in allograft response, we isolated CD8^+^ T cells from WT B6, *Batf*^−/−^, and *Batf*^−/−^ *Batf3*^−/−^ mice and adoptively transferred them into syngeneic *Rag1*^−/−^mice (0.5 × 10^6^ cells per mouse). On the second day, skin allografts from BALB/c mice were transplanted into *Rag1*^−/−^ mice and allograft survival was assessed (Fig. 2A). As expected, *Rag1*^−/−^ mice that received CD8^+^ T cells from WT and *Batf3*^−/−^ mice readily rejected the skin allograft [median survival time (Blank et al. 2019) = 15.5 days and 17 days; n = 6 per group]. In contrast, recipients who received *Batf*^−/−^ CD8^+^ T cells presented with long-term allograft survival (MST = 55 days; n = 6). Interestingly, B6. *Rag1*^−/−^ recipients who received *Batf*^−/−^ *Batf*3^−/−^ CD8^+^ T cells no longer exhibited rejection of the skin allografts from BALB/c mice (MST > 100 days; n = 6) (Fig. 2B, p < 0.001). As shown in Fig. 2C, skin graft rejection was observed in recipients of control CD8^+^ T cells (left image), while the skin graft on the recipient receiving *Batf*^−/−^ *Batf*3^−/−^ CD8^+^ T cells showed normal skin appearance (middle and right images). In representative hematoxylin- and eosin-stained images, skin grafts from the control group demonstrated severely damaged skin structures and massive inflammatory cell infiltration on day 14 after skin grafting. In contrast, skin grafts from the *Batf*^−/−^ *Batf*3^−/−^ CD8^+^ T cell group showed intact skin structure and minimal inflammatory cell infiltration (Fig. 2D). The control CD8^+^ T cell group on day 14 after skin transplantation showed a higher skin transplantation PR score than the *Batf*^−/−^ *Batf*3^−/−^ CD8^+^ T cell group on day 14 and 100 after skin transplantation (Fig. 2E, p < 0.01).

### BATF and BATF3 deficiency attenuates the CD8+ T cell allo-response

**Fig. 3:**
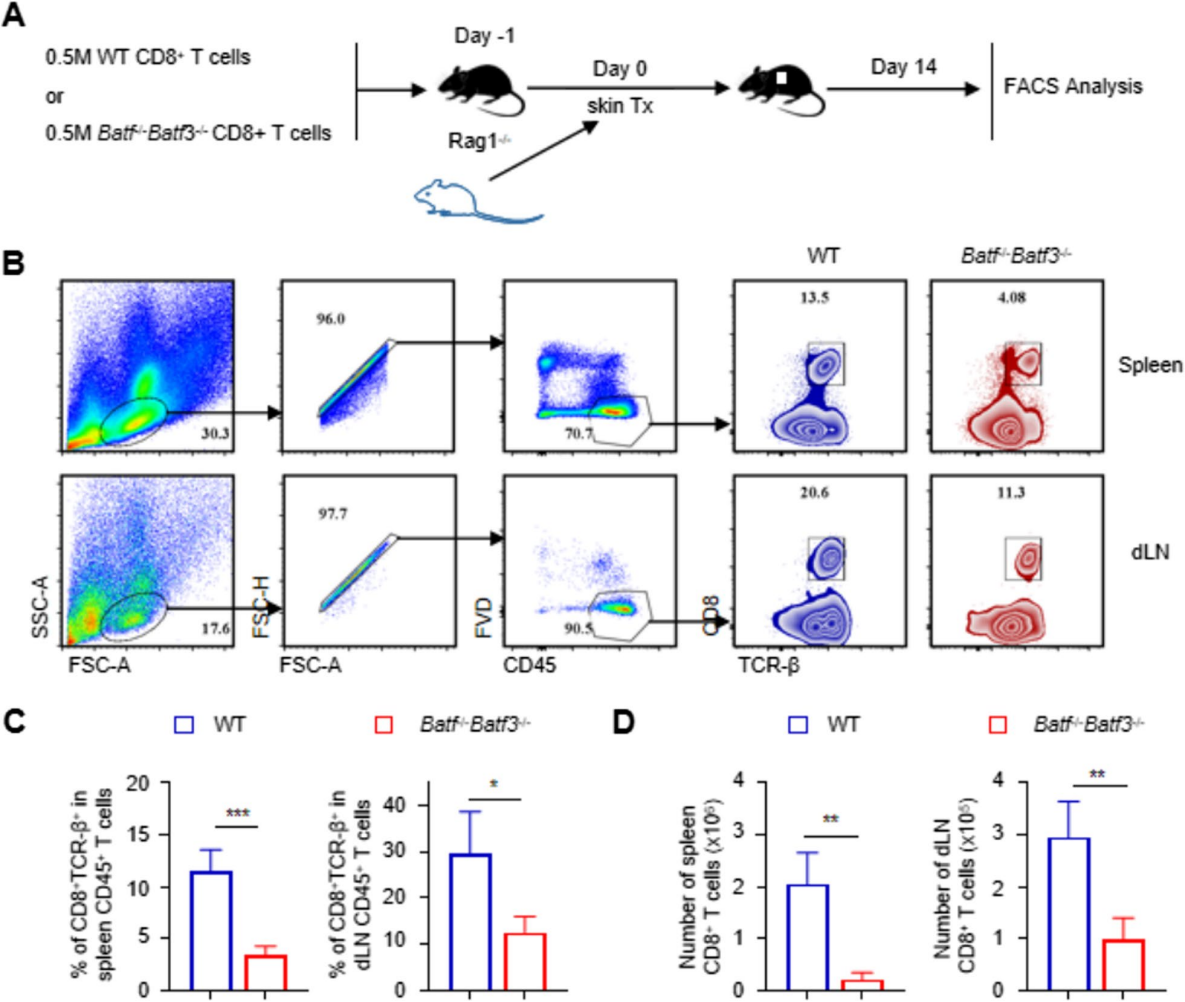
BATF and BATF3 deficiency attenuates the CD8+ T cell allo-response. **A** WT and Batf−/−Batf3−/− T cells adoptively transferred to Rag1−/− recipients were analyzed on days 14 after skin transplantation. **B**, **C** Representative contour plots and bar graphs of % CD8+ TCR-β+ in spleen and dLNs CD45+ cells. **D** Representative bar graph shows number of spleen and dLNs CD8+ T cells in recipients. *p < 0.05; **p < 0.01,***p < 0.001

To better understand the dynamics in the number of CD8^+^ T cells in recipients after transplantation, we harvested spleens and drained lymph nodes from *Rag1*^−/−^ recipients transferred with WT CD8^+^ T cells and *Batf*^−/−^ *Batf*3^−/−^ CD8^+^ T cells 14 days after skin transplantation. Cell suspensions were prepared for flow cytometry analysis. At day 0 after separation, the cells have comparability (Additional file 2: Figure S2). However at day 14, we found that the proportion of CD8^+^ TCR^+^ T cells in the spleen and dLNs of *Rag1*^−/−^ mice transferred with *Batf*^−/−^ *Batf*3^−/−^ CD8^+^ T cells were lower than in the control group. As shown in Fig. 3D, the number of CD8^+^ cells in the spleen and dLNs of *Batf*^−/−^ *Batf*3^−/−^ recipients was much lower than in WT recipients. Moreover, the proliferative function of CD8^+^ T cells lacking BATF and BATF3 was significantly reduced. For further verification, after seperation and purification, harvested CD8^+^ T cells were stimulated by anti-CD3 and anti-CD28 Ab in vitro for 48h. *Batf*^−/−^ *Batf3*^−/−^ CD8^+^ T cells showed lower activation and proliferation ability (Additional file 3: Figure S3).

### BATF and BATF3 deficiency inhibits functional differentiation of effector CD8+ T cells

**Fig. 4:**
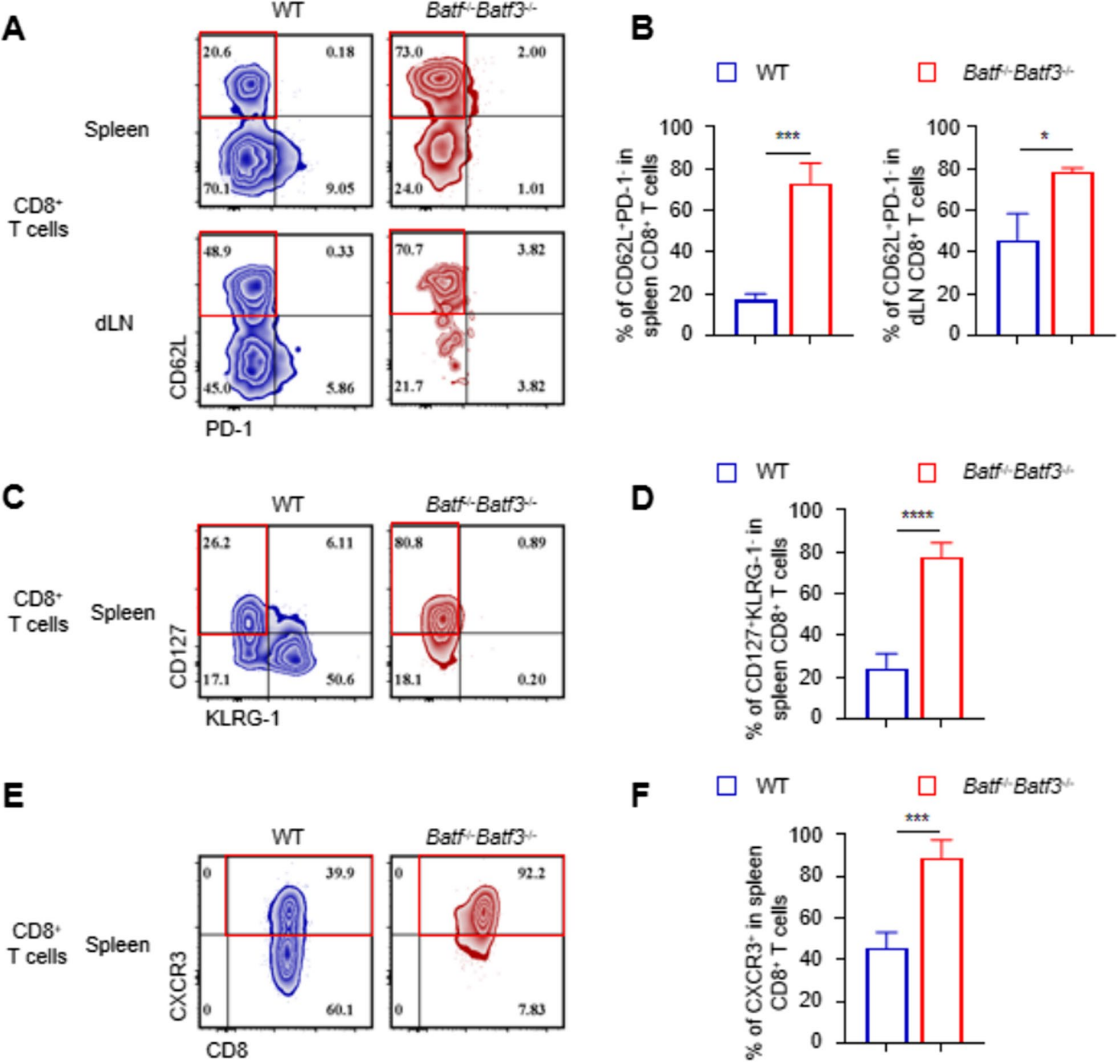
BATF and BATF3 deficiency inhibits functional differentiation of effector CD8+ T cells. **A**, **B** Representative contour plots and bar graphs showing the % of CD62L+PD-1-cells in co- transferred WT CD8+ T cells and Batf−/−Batf3−/−CD8+ T cells 14 days after skin transplants. Data are presented as means ± SD (n = 5) and are representative of three independent experiments. **, p < 0.01, ***, p < 0.001; unpaired Student’s t- test. **C**, **D** Representative contour plots and bar graphs show the % of CD127+KLRG-1-cells in spleen CD8+ T cells, ****, p < 0.0001. **E**, **F** FACS plots and bar graphs showing CXCR3 expression by co-transferred WT and batf−/− batf3−/− CD8+ T cells from skin transplant recipients. ***, p < 0.001

It has long been established that the in vivo differentiation status of CD8^+^ T cells is critical for graft rejection. Naïve or memory T cells differentiate into effector T cells, which participate in graft rejection. To observe the phenotypic changes in CD8^+^ T cells transferred into *Rag1*^−/−^ mice after skin transplantation, we analyzed the recipient's spleen and dLNs 14 days after surgery. Compared with the control group, most of the spleen *Batf*^−/−^ *Batf*3^−/−^ CD8^+^ T cells remained at the CD62L^+^PD-1- CD8^+^ T cell stage, and the CD62L^+^PD-1- CD8^+^ T cell proportion in the dLNs of the *Batf*^−/−^ *Batf*3^−/−^ recipients was higher than the control group (Fig. 4 A, B). As shown in Figs. 4C, 4D, the CD8^+^ T cells in the spleen of the control group could differentiate into CD127-KLRG-1^+^ effector CD8 T cells, while most spleen CD8^+^ cells in the *Batf*^−/−^ *Batf*3^−/−^ CD8^+^ T group were CD127^+^KLRG- cells. CXCR3 is a characteristic surface molecule of naïve T cells. Fourteen days after transplantation, 92.2% of transferred *Batf*^−/−^ *Batf*3^−/−^ CD8^+^ T cells were CXCR3^+^CD8^+^ T cells, while transferred WT CD8^+^ T cells only partially expressed CXCR3 (Fig. 4E, F). The above findings substantiate that BATF and BATF3 deficiencies affect naïve CD8^+^ T cells that differentiate into terminal effector cells.

### BATF and BATF3 deficiency attenuates early functional expression of effector CD8+ T cells

**Fig. 5:**
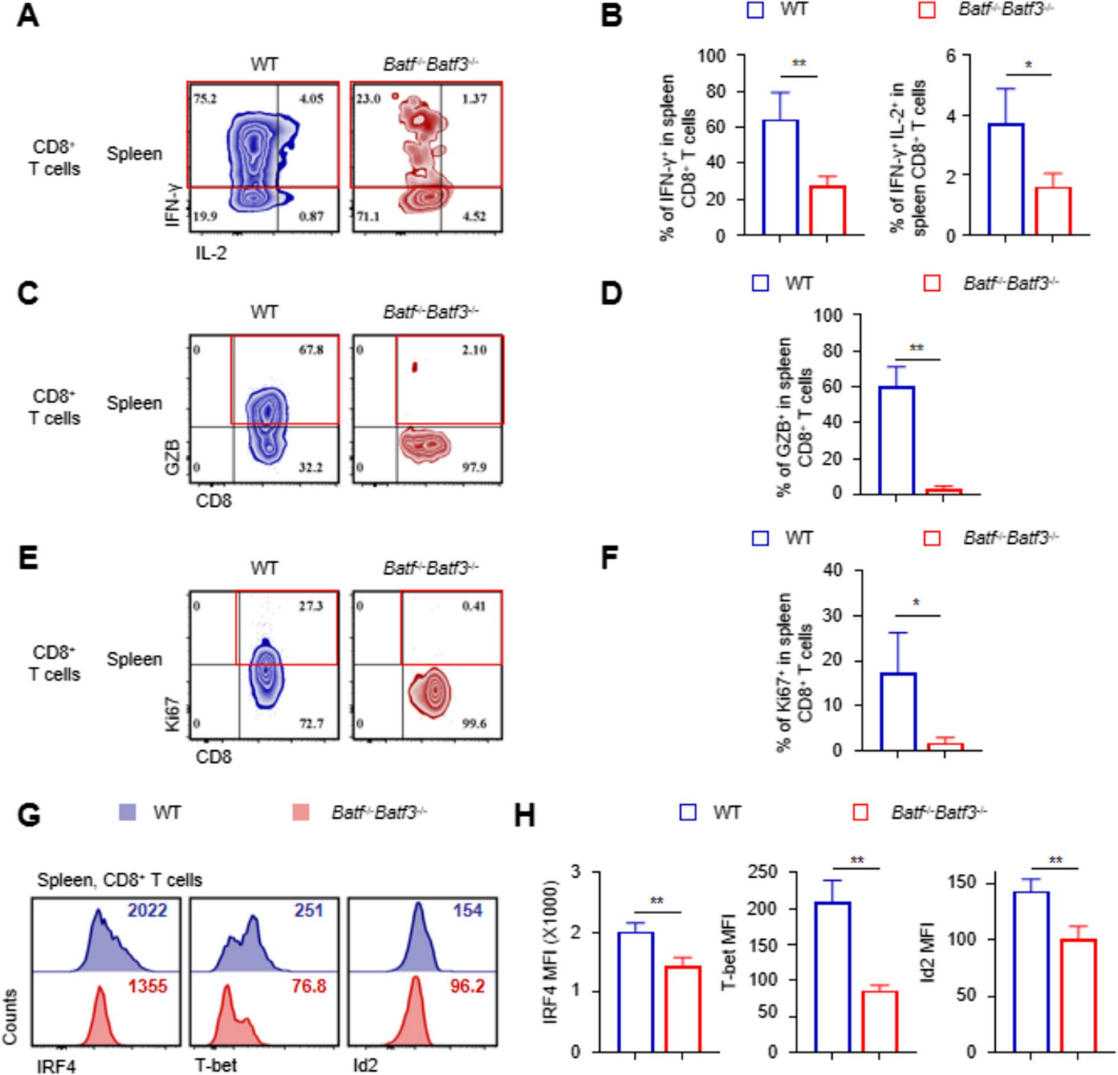
BATF and BATF3 deficiency attenuates early functional expression of effector CD8+ T cells. **A** B6.Rag1-/-recipients were adoptively transferred with either batf−/− batf3−/− or control CD8+ T cells, followed by BALB/c skin transplantation. Adoptively transferred CD8+ T cells in spleens were analysed by flow cytometry at day 14 post-skin grafting. **A**, **B** Representative contour plots and bar graphs show % of IFN-γ+ and IFN-γ+IL-2+CD8+ T cells in spleen CD8+ T cells. **C**–**F** Contour plots were gated on the living transferred CD8+ T cells in spleens. Representative contour plots and bar graphs show % GZB+CD8+ (**C**, **D**) and Ki67+CD8+ (**E**, **G**) cells in spleen CD8+ T cells. *, p < 0.05; **, p < 0.01. **G**, **H** Representative fluorescence intensity distribution maps and MFI bar graphs of IRF4, T-bet and Id2 in spleen CD8 + T cells. **p < 0.01 by unpaired Student’s t-test

Current evidence suggests that effector CD8^+^ T cells function by secreting effector molecules, such as IL-2, IFNγ, TNFα, and granzymes. To examine the effector function of transferred CD8^+^ T cells after skin transplantation, we analyzed recipient spleen cells 14 d post-BALB/c skin grafting. Compared with the control CD8^+^ T cells in spleens, the transferred *Batf*^−/−^ *Batf*3^−/−^ CD8^+^ T cells exhibited significantly decreased levels of effector cytokines, especially IFN-γ and IL-2 (Fig. 5A, B). Meanwhile, in *Rag1*^−/−^ mice after transplantation, *Batf*^−/−^ *Batf*3^−/−^ CD8^+^ T cells did not produce GzB, whereas 67.8% of WT CD8^+^ T cells secreted GzB (Fig. 5C, D). Moreover, the expression levels of the proliferation marker Ki67 in transferred *Batf*^−/−^ *Batf*3^−/−^ CD8^+^ T cells were significantly reduced compared to the control CD8^+^ T cells (Fig. 5E, F). These findings suggest that *Batf*^−/−^ *Batf*3^−/−^ CD 8^+^ T cells lost the ability to produce effector molecules and express Ki67 in transplant recipients. On the other hand, several key transcription factors are important regulators in the differentiation of effector CD8^+^ T cells. The reduction in IRF4, T-bet and Id2 expression were significant in transferred Batf−/−Batf3−/−CD8+ T cells (Fig. 5G, H).

### BATF and BATF3 deficiency led to long-term inhibition of effector CD8+ T cells

**Fig. 6:**
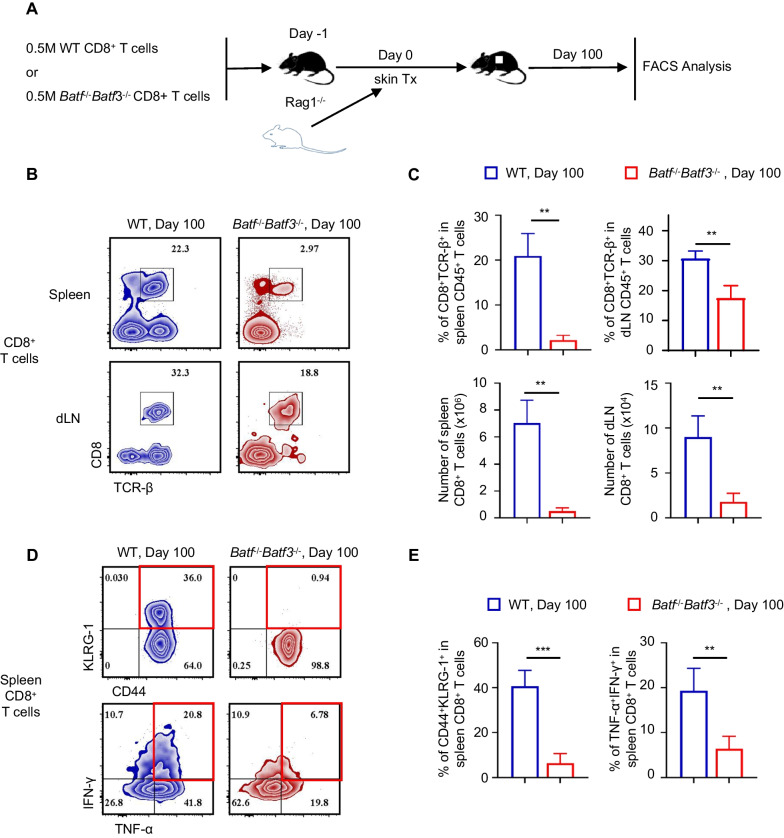
BATF and BATF3 deficiency led to long term inhibition of effector CD8 + T cells. **A** At day 100 post-BALB/c skin grafting, the adoptively transferred batf−/−batf3−/− and control CD8+ T cells in B6.Rag1−/− recipients were analysed. **B** Representative contour plots show percentage of the CD8+TCR-β+ cells in spleen and dLNs CD8+ cells. **C** Bar graphs show percentage a CD8+TCR-β+ cells in spleen and dLNs CD45+ cells and number of CD8+ TCR-β + cells in spleen and dLNs. **D**, **E** % of CD44+KLRG-1+ cells and TNF-α+IFN-γ+ cells in spleen CD8+ T cells. Data in **C**, **E** are shown as mean ± SD and are from one experiment that is representative of 2 independent experiments. ns, p > 0.05; *p < 0.05; **p < 0.01; ***p < 0.001 by unpaired Student’s t-test

Effector CD8^+^ T cells are important indicators for evaluating the long-term effects of BATF and BATF3 on transplantation. In the present study, after CD8^+^ T cells were transferred, all control group recipients exhibited graft rejection, while the skin graft was successful in the *Batf*^−/−^ *Batf*3^−/−^ group. Therefore, we analyzed adoptively transferred CD8^+^ T cells in the spleens by flow cytometry at day 100 post-grafting. Figures 6B, C show the proportion and abundance of CD8^+^ T cells in the spleen and dLNs from the *Batf*^−/−^ *Batf*3^−/−^ and control groups. At 100 days after transplantation, both the proportion and abundance of CD8^+^ T cells in the spleen and dLNs of *Batf*^−/−^ *Batf*3^−/−^ mice were significantly lower than in the control group. Interestingly, the number and abundance of CD8^+^ T cells in the spleen of *Batf*^−/−^ *Batf*3^−/−^ recipients decreased 100 days after transplantation compared with day 14, while the proportion of CD8^+^ T cells in dLNs increased. *Batf*^−/−^ *Batf*3^−/−^ CD8^+^ T cells did not differentiate into CD44^+^KLRG-1^+^CD8^+^T cells. In contrast, 36.0% of CD8^+^ T cells in the control group showed high CD44 and KLRG expression. Moreover, the percentages of TNF-α^+^IFN-γ^+^ in spleen CD8^+^ T cells of the *Batf*^−/−^ *Batf*3^−/−^ group were significantly lower than control CD8^+^ T cells (Fig. 6 D, E).

### BATF and BATF3 deficiency prompts the generation of exhausted CD8+ T cells

**Fig. 7:**
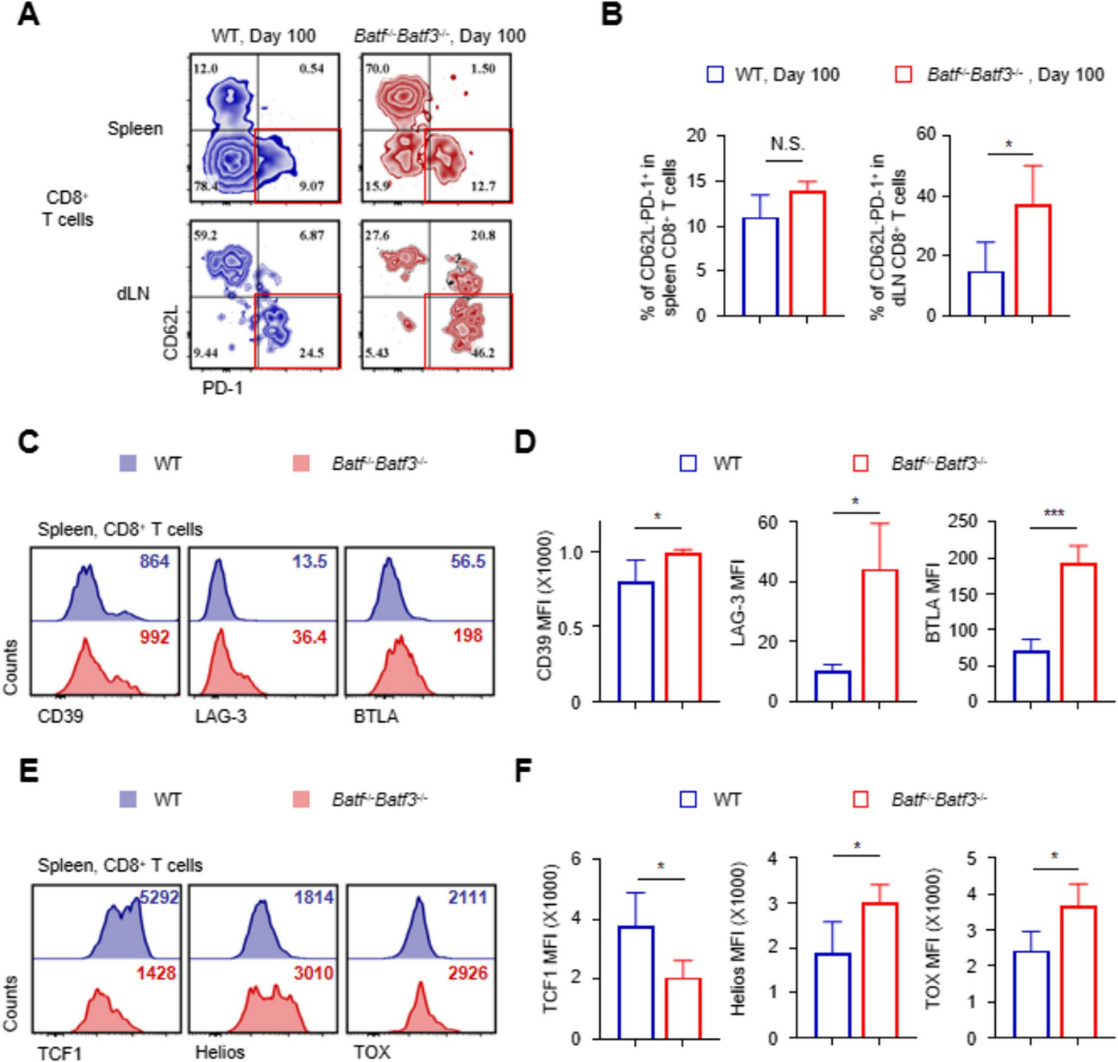
BATF and BATF3 deficiency prompts the generation of exhausted CD8+ T cells. **A**, **B** Representative contour plots and the bar graph show % of CD62L^−^PD-1^+^ in spleen and dLNs CD8+ T cells. **C**, **D** Representative fluorescence intensity distribution maps and MFI bar graphs of CD39, LAG-3 and BTLA in spleen CD8^+^ T cells. **E**, **F** Representative fluorescence intensity distribution maps and MFI bar graphs of TCF1,Helios and TOX in spleen CD8 ^+^ T cells. *p < 0.05; ***p < 0.001 by unpaired Student’s t-test

The roles of BATF and BATF3 in mediating the generation of exhausted CD8^+^T cells are crucial in transplantation. At day 100 post-grafting, T cell exhaustion induced an increase in inhibitory receptors, while the abundance of CD62L-PD-1^+^ in spleen CD8^+^ T cells was not significantly different between the two groups. Moreover, the abundance of CD62L-PD-1^+^ in dLN CD8^+^ T cells was significantly lower than in control CD8^+^ T cells (Fig. 7A, B). Moreover, the expression of other inhibitory receptors such as CD39, LAG-3 and BTLA was increased on Batf^−/−^ Batf3^−/−^ CD8+ T cells compared with the control group (Fig. 7C, D). It has been reported that transcription factors play a critical role in regulating CD8+ T exhaustion. We analyzed the expression of several transcription factors and found the mean fluorescence intensities of Helios and TOX in Batf^−/−^ Batf3^−/−^ CD8+ T cells were significantly higher than in control groups, while the mean fluorescence intensities of TCF-1 were lower (Fig. 7E, F). These findings suggest that BATF and BATF3 deficiencies in CD8^+^ T cells lead to long-term defects in effector function and CD8^+^ T cell exhaustion, which promotes long-term allograft survival.

## Discussion

T cells play a central role in allograft rejection, and CD8^+^ T cells with cytotoxic effects are key players mediating allograft injury during rejection (Yap et al. 2015). However, the exact transcriptional regulatory mechanism of CD8^+^ T cells in allograft rejection remains unclear. In this study, we found that the expression of BATF and BATF3, members of the AP1 family, was positively correlated with the degree of activation of CD8^+^ T cells. During co-transfer experiments, we found that *Batf*^−/−^*Batf*3^−/−^ CD8^+^ T cells showed weak activation and proliferation during the early stages after skin transplantation and failed to produce proinflammatory cytokines and cytotoxic molecules. Subsequently, long-term graft survival was observed. It has been reported that low activation levels of CD8^+^ T cells can last for 100 days after skin transplantation, and these cells display an exhausted phenotype with increased expression of inhibitory molecules. Taken together, our findings suggest that BATF and BATF3 deficiencies in CD8^+^ T cells disrupt the balance between effector and exhausted subsets, prolonging graft survival.

Generally, recipient naive CD8^+^ T cells recognize intact MHC class I alloantigens on the surface of the donor or recipient-self antigen-presenting cells (APCs) through direct or indirect pathways and exert cytotoxic effects when activated. (Rascio et al. 2021). However, late after transplantation, allograft antigen persistent stimulation and activation signals drive another distinct T-cell differentiation program. T cells retain suboptimal but crucial functions to reach a “reconciliation” with allografts, called exhausted T cells (Sanchez-Fueyo and Markmann 2016). Unlike T-bet and TCF1, Eomesodermin (EOMES) is highly expressed in PD1^high^TIM3^+^ exhausted T cells (Tex)(Wherry and Kurachi 2015).

BATF is a critical transcription factor for the differentiation of effector CD8^+^ T cells. An increasing body of evidence suggests that BATF-deficient CD8^+^ T cells display impaired effector phenotypes against cancer and infections (Kurachi et al. 2014; Li et al. 2022). Consistently, we found that BATF/BATF3-deficient CD8^+^ T cells failed to differentiate into effectors and could not produce proinflammatory cytokines and cytotoxic molecules (IL2, IFN-γ, TNFα, and GzB). It is worth mentioning that although persistently high PD1 levels are regarded as an exhaustion signal (Blank et al. 2019), mild PD1 elevation is often observed during the early activation of CD8^+^ T cells (Ahn et al. 2018). On day 14 after transplantation, PD1, an inhibitory receptor, was used to determine whether CD8^+^ T cells were activated and whether *Batf*^−/−^*Batf*3^−/−^ CD8^+^ T cells remained inactivated or naïve. On day 100, PD1 exhibited high expression levels, highlighting its value as a classical marker of exhausted CD8+ T cells.

BATF3 is regarded as the master transcription factor of cDC during the cross-presentation process (Hildner et al. 2008; Duong et al. 2022). Recent evidence suggests that BATF3-deficient CD8^+^ T cells exhibit defects in T cell memory formation, reduced proliferation rates, and increased cell death following viral or bacterial infections (Ataide et al. 2020). In a previous study, we established that BATF3 could compensate for the function of BATF, and BATF3 expression could be increased during BATF deficiency. Simultaneous double knock-out of BATF and BATF3 can further enhance the inhibitory effect on CD4^+^ T cell activation compared to single knock-out (Wang et al. 2022; Lee et al. 2019). This finding was observed in CD8^+^ T cells in the early effector and late exhausted phases.

BATF interacts with several key transcription factors to regulate the differentiation of effector CD8^+^ T cells. In this respect, IRF4 plays a critical role in the early activation of T-cells. CD8^+^ T cells exhibit a limited ability to differentiate into effector T cells during IRF4 deletion (Zou et al. 2020). A recent study established a chronic infection model and revealed that memory subsets’ proliferation rates and function were impaired when IRF4 was knocked out while exhausted subsets emerged (Man et al. 2017). Overwhelming evidence substantiates that BATF and IRF4 work synergistically to regulate CD8^+^ T cell differentiation (Man et al. 2017; Grusdat et al. 2014). Mechanistically, BATF forms a trimeric complex, an AP1–IRF composite element with Jun family proteins and IRF4, which co-regulates gene transcription (Li et al. 2012). TCF-1 and T-bet are essential for T-cell differentiation and aging. Generally, exhausted T cells exhibit loss of T-bet and TCF-1 expression (Blank et al. 2019; Wherry and Kurachi 2015), and their binding sites largely overlap with BATF(Tsao et al. 2022; Scott-Browne et al. 2016). Moreover, Id2 is required for the terminal differentiation of effector CD8+ T cells(Omilusik et al. 2018). TOX and Helios mediate the generation of exhausted CD8+ T cells(Khan et al. 2019; Pieren et al. 2022). Overall, this study provided hitherto undocumented evidence of the potential relationship between BATF and these transcription factors. Id2, TOX and Helios were differentially expressed in the absence of BATF and BATF3. It is highly conceivable that BATF regulates the transcription of T cells by altering the accessibility of these factors.

A major shortcoming of our study was that no CD8^+^ T cell-specific knock-out mice were used. Accordingly, the interference of total knock-out BATF and BATF3 cannot be excluded since they play a critical role in Tfh, Treg, and Th cells, among others (Delacher et al. 2020; Liu et al. 2013; Shetty et al. 2022). Moreover, in vitro further mechanism exploration of the affect of BATF and BATF3 on CD8^+^ T cells remains future study.

## Conclusion

In this study, wild-type CD8^+^ T cells, BATF-deficient (*Batf*^−/−^) CD8^+^ T cells, and CD8^+^ T cells deficient in both BATF and BATF3 (*Batf*^−/−^ *Batf*3^−/−^) were transferred to B6.Rag1^−/−^ mice, which received skin allografts from BALB/c mice. BATF and BATF3-deficient mice exhibited limited rejection of major histocompatibility complex (MHC)-mismatched skin allografts without tolerating therapies. Mechanistically, BATF and BATF3 deletion affected the differentiation of effector CD8^+^ T cells and mediated the exhaustion of CD8^+^ T cells. Our findings substantiate that BATF proteins have huge prospects as a potential therapeutic approach to achieving skin transplant acceptance.

### Supplementary Information

**Additional file 1: Figure S1.** Representative fluorescence intensity distribution maps of CD8 in spleen cells before and after seperated by Dynabeads Untouched Mouse CD8 Cells Kit.

**Additional file 2: Figure S2.** Representative contour plots and the bar graph show % of CD25+CD69+ in just seperated CD8+ T cells. N.S., p > 0.05 by unpaired Student’s t-test.

**Additional file 3: Figure S3.** (A)Representative contour plots and the bar graph show % of Ki67+ in CD8+ T cells after stimulation for 48h.; Representative fluorescence intensity distribution maps of CFSE and bar graph of % proliferated cells in CD8+ cells after stimulation for 48h. (B) Representative contour plots and the bar graph show % of CD25+CD69+ in living seperated CD8+ T cells after stimulation for 48h. ***, p < 0.001; ****, p < 0.0001 by unpaired Student’s t-test.

## Data Availability

The datasets during and/or analysed during the current study available from the corresponding author on reasonable request.

## Abbreviations

TCR: T cell receptor
WT: Wild-type
BATF: Basic leucine zipper ATF-like transcription factor
IRF4: Interferon regulatory factor 4
MHC: Major histocompatibility complex
KLRG1: Killer cell lectin like receptor G1
TCF1: T cell factor 1
Id2: Inhibitor 2 of DNA binding
