# Supplementary figures and images for: BATF and BATF3 deficiency alters CD8+ effector/exhausted T cells balance in skin transplantation
Source: Mol Med. 2024 Jan 31;30:16. doi: 10.1186/s10020-024-00792-0 (PMC10832090; doi:10.1186/s10020-024-00792-0)

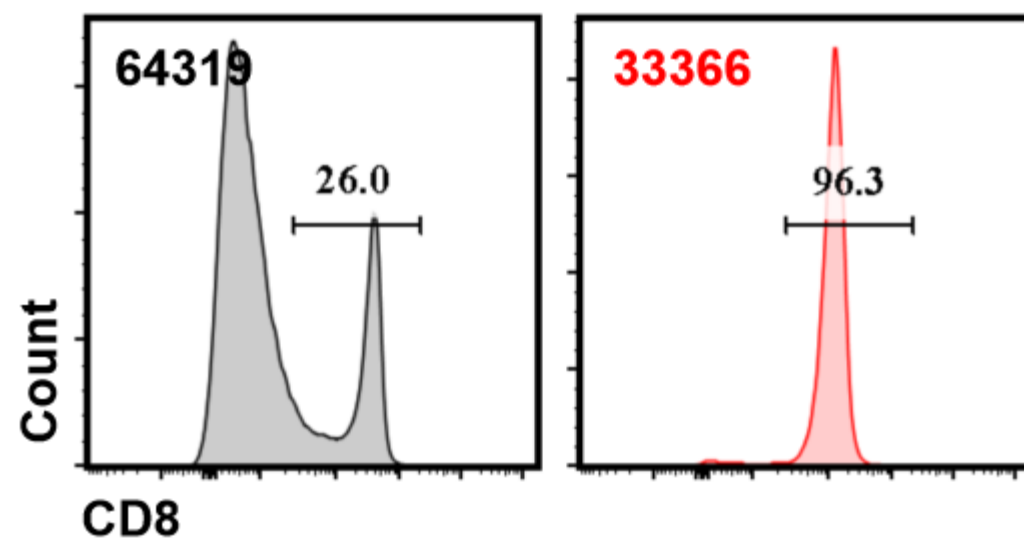

Supplement: Supplementary file 1 — Additional file 1: Figure S1. Representative fluorescence intensity distribution maps of CD8 in spleen cells before and after seperated by Dynabeads Untouched Mouse CD8 Cells Kit. [file 10020_2024_792_MOESM1_ESM.pdf]

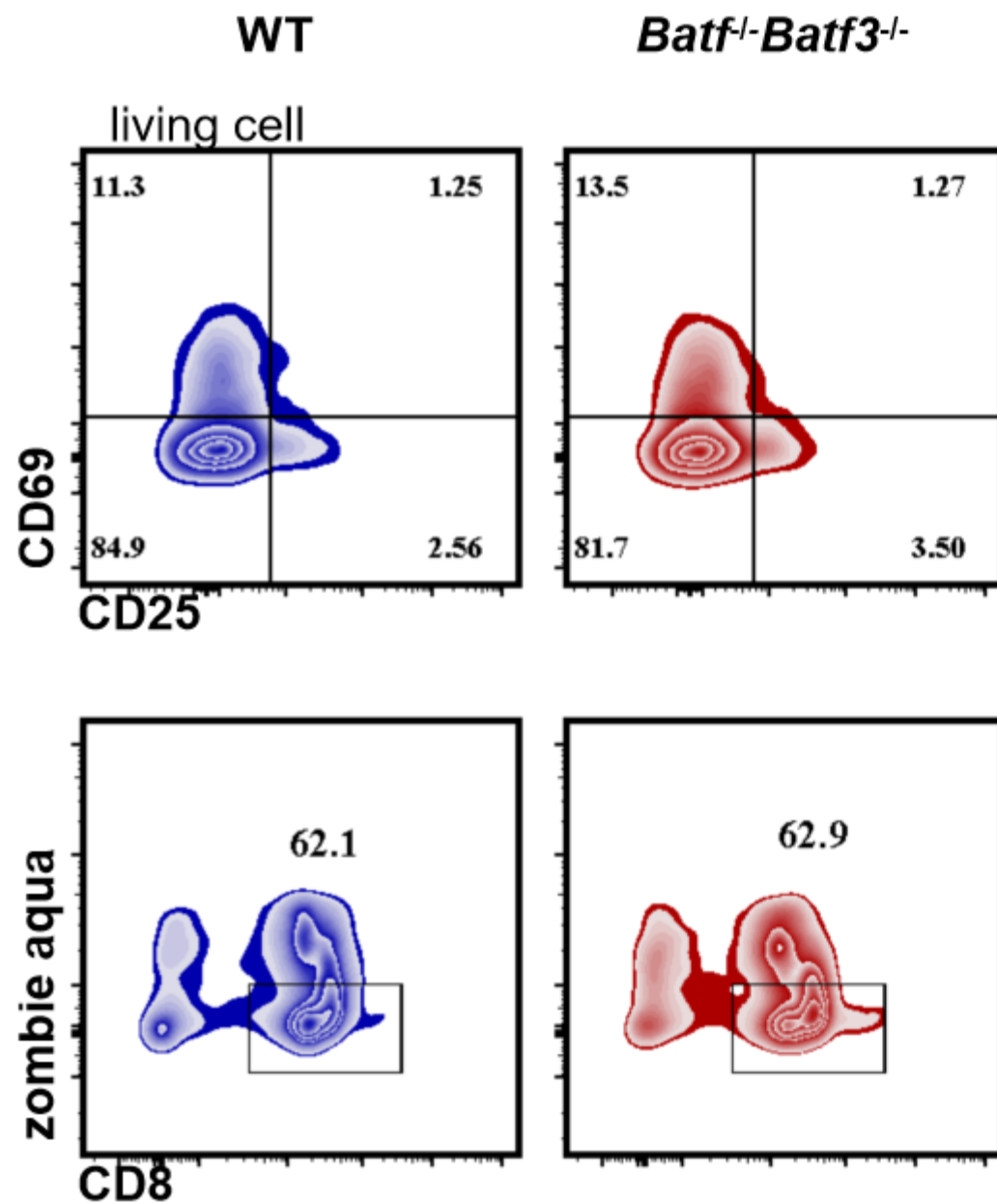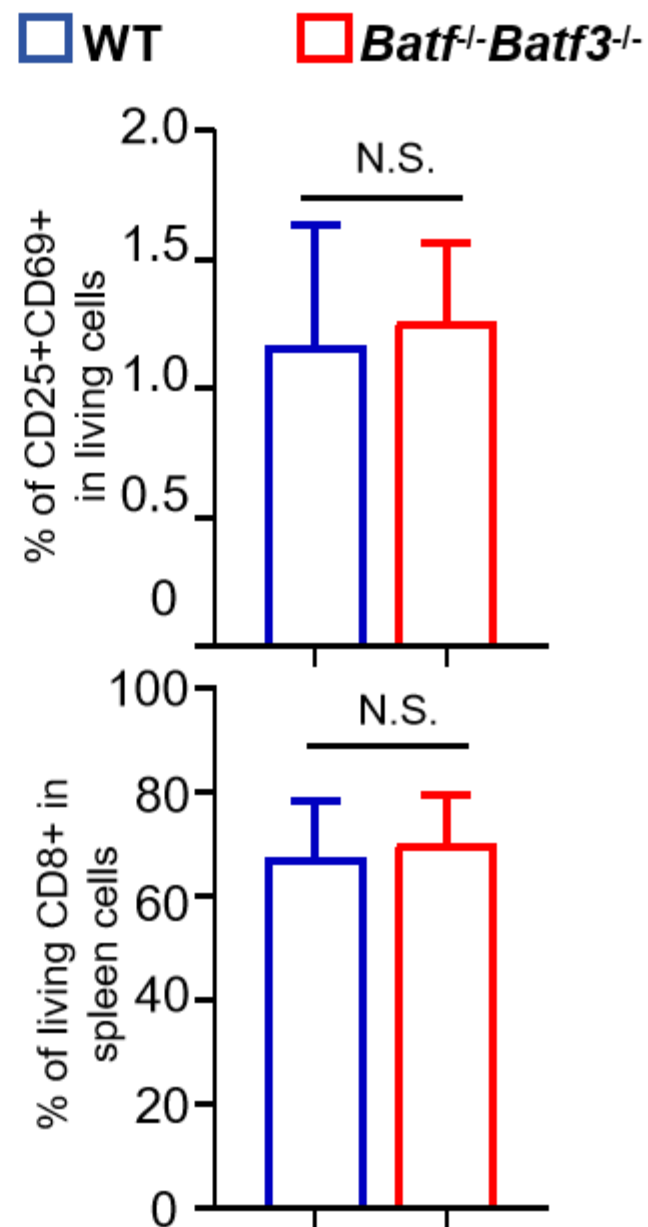

Supplement: Supplementary file 2 — Additional file 2: Figure S2. Representative contour plots and the bar graph show % of CD25+CD69+ in just seperated CD8+ T cells. N.S., p > 0.05 by unpaired Student’s t-test. [file 10020_2024_792_MOESM2_ESM.pdf]

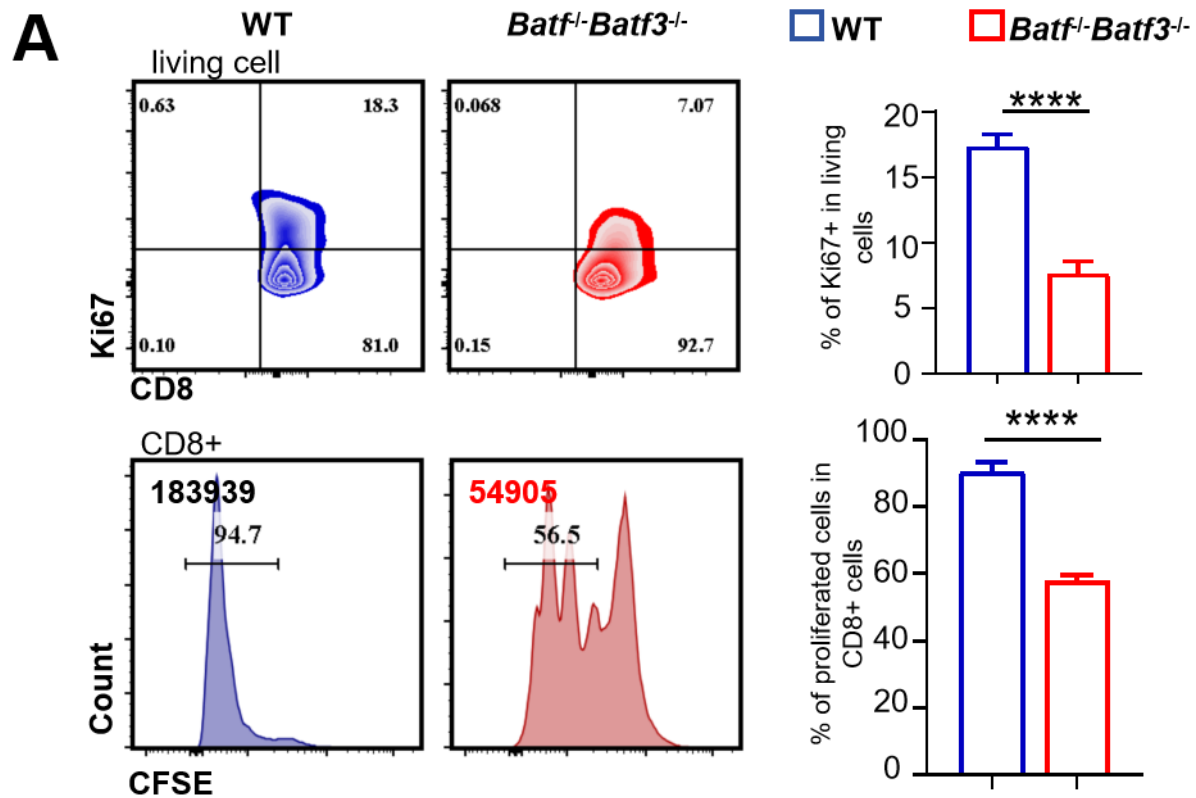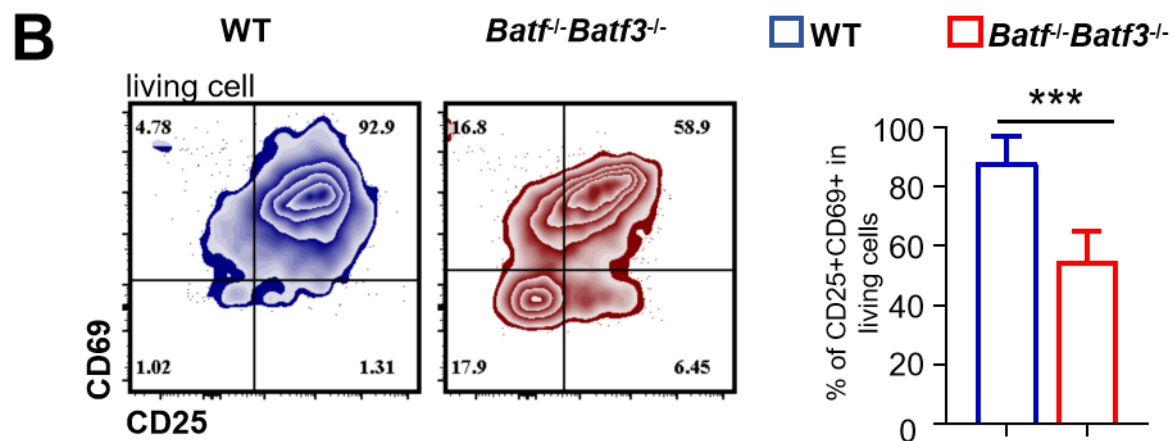

Supplement: Supplementary file 3 — Additional file 3: Figure S3. (A)Representative contour plots and the bar graph show % of Ki67+ in CD8+ T cells after stimulation for 48h.; Representative fluorescence intensity distribution maps of CFSE and bar graph of % proliferated cells in CD8+ cells after stimulation for 48h. (B) Representative contour plots and the bar graph show % of CD25+CD69+ in living seperated CD8+ T cells after stimulation for 48h. ***, p < 0.001; ****, p < 0.0001 by unpaired Student’s t-test. [file 10020_2024_792_MOESM3_ESM.pdf]
